# Applying eHealth for Pandemic Management in Saudi Arabia in the Context of COVID-19: Survey Study and Framework Proposal

**DOI:** 10.2196/19524

**Published:** 2020-11-26

**Authors:** Abdullah Alsharif

**Affiliations:** 1 Department of Management Information Systems College of Business Administration-Yanbu Taibah University Medinah Saudi Arabia

**Keywords:** COVID-19, eHealth framework, infectious disease, pandemic, eHealth, public health

## Abstract

**Background:**

The increased frequency of epidemics such as Middle East respiratory syndrome, severe acute respiratory syndrome, Ebola virus, and Zika virus has created stress on health care management and operations as well as on relevant stakeholders. In addition, the recent COVID-19 outbreak has been creating challenges for various countries and their respective health care organizations in managing and controlling the pandemic. One of the most important observations during the recent outbreak is the lack of effective eHealth frameworks for managing and controlling pandemics.

**Objective:**

The aims of this study are to review the current National eHealth Strategy of Saudi Arabia and to propose an integrated eHealth framework that can be effective for managing health care operations and services during pandemics.

**Methods:**

A questionnaire-based survey was administered to 316 health care professionals to review the current national eHealth framework of Saudi Arabia and identify the objectives, factors, and components that are key for managing and controlling pandemics. Purposive sampling was used to collect responses from diverse experts, including physicians, technical experts, nurses, administrative experts, and pharmacists. The survey was administered at five hospitals in Saudi Arabia by forwarding the survey link using a web-based portal. A sample population of 350 was achieved, which was filtered to exclude incomplete and ineligible samples, giving a sample of 316 participants.

**Results:**

Of the 316 participants, 187 (59.2%) found the current eHealth framework to be ineffective, and more than 50% of the total participants stated that the framework lacked some essential components and objectives. Additional components and objectives focusing on using eHealth for managing information, creating awareness, increasing accessibility and reachability, promoting self-management and self-collaboration, promoting electronic services, and extensive stakeholder engagement were considered to be the most important factors by more than 80% of the total participants.

**Conclusions:**

Managing pandemics requires an effective and efficient eHealth framework that can be used to manage various health care services by integrating different eHealth components and collaborating with all stakeholders.

## Introduction

### Background

eHealth can be basically defined as the application of information and communications technology (ICT) in the health care sector [1]. eHealth is considered to be one of the major developments of the past few decades and has revolutionized the operation of health care services. Various studies [2-4] have indicated the positive impact of eHealth approaches in improving health care service delivery, minimizing operational costs, increasing process efficiency, and most importantly, managing health care information. Focusing on the concept of “application” derived from the definition in [1], the success of eHealth depends on how it is applied and managed in different areas of health care operation. To improve the process of eHealth implementation, different frameworks were developed by different countries (eg, Austria in the European Union) and health care organizations according to their needs and specifications [5,6]. However, these frameworks and documents mainly focus on examples and present outlines (high level designs); meanwhile, they offer very little information to guide the process of development [1]. A few studies [7-9] have attempted to provide frameworks for strategic planning and implementation of eHealth strategies. However, these studies are mostly related to normal health care operations. In a few studies [10,11], frameworks were developed for health care management during pandemics; however, these studies are limited in scope, focusing on frameworks for assessing preparedness and readiness but not for managing health care operations during pandemics. These studies focused on readiness strategies, such as resource management, finances, and vaccination; none of them highlighted an operational framework for pandemics that includes eHealth approaches or stakeholders’ roles and responsibilities. In addition, these studies were distributed across different regions and may not be applicable in all cases.

National eHealth frameworks have been formulated based on the vision and selected objectives targeting health care management and dissemination of general health care services [6,12]. These frameworks may not be effective in dealing with pandemics, which may require sudden changes in health care policies, strategies, and information management. For instance, various myths and pieces of misinformation about COVID-19 were observed to be circulating over the internet and proved to be challenging for governments and health care officials to address [13]. In addition, pandemics have been identified more frequently in recent years, including severe acute respiratory syndrome (SARS), H7N9 influenza, Zika virus, Ebola virus, Middle East respiratory syndrome (MERS), and COVID-19. Considering these unexpected risks and other health care challenges, sudden changes in health care policies and strategies may be inevitable. In these conditions, creating awareness among the public, disseminating legitimate information, and making changes to health care services such as access and delivery are aspects of eHealth that can be useful in managing pandemics. eHealth can be one of the most effective operations during pandemics, as it can enable remote management of health care operations and services. Focusing on the gaps identified in relation to the management of health care operations during pandemics, and considering these challenges and the possibilities of using eHealth approaches to pandemic management, the aims of this paper are to review the existing national eHealth framework of Saudi Arabia and to propose an integrated eHealth framework for managing pandemics in Saudi Arabia.

### Literature Review

According to the World Health Organization (WHO) [14], of its 194 member countries, only 58% have an eHealth strategy, and only 55% have established legislation in relation to eHealth data. The frameworks developed by these countries were based on their needs and considered various contexts, such as finance, resources, and technological capabilities. Because few countries have an eHealth framework and proper eHealth legislation, it can be challenging for countries to coordinate and manage health care operations during pandemics, when there is a need for collaboration among countries to manage the pandemic. However, it is equally important to manage health care operations internally and control the rapid spread of disease and infection during pandemics. Different health care components may need to be effectively managed during pandemics. Accordingly, this section provides a review of the literature regarding various components of eHealth frameworks and their applicability in pandemics according to the guidelines prescribed by the WHO [15].

#### Stakeholders

**Figure 1:**
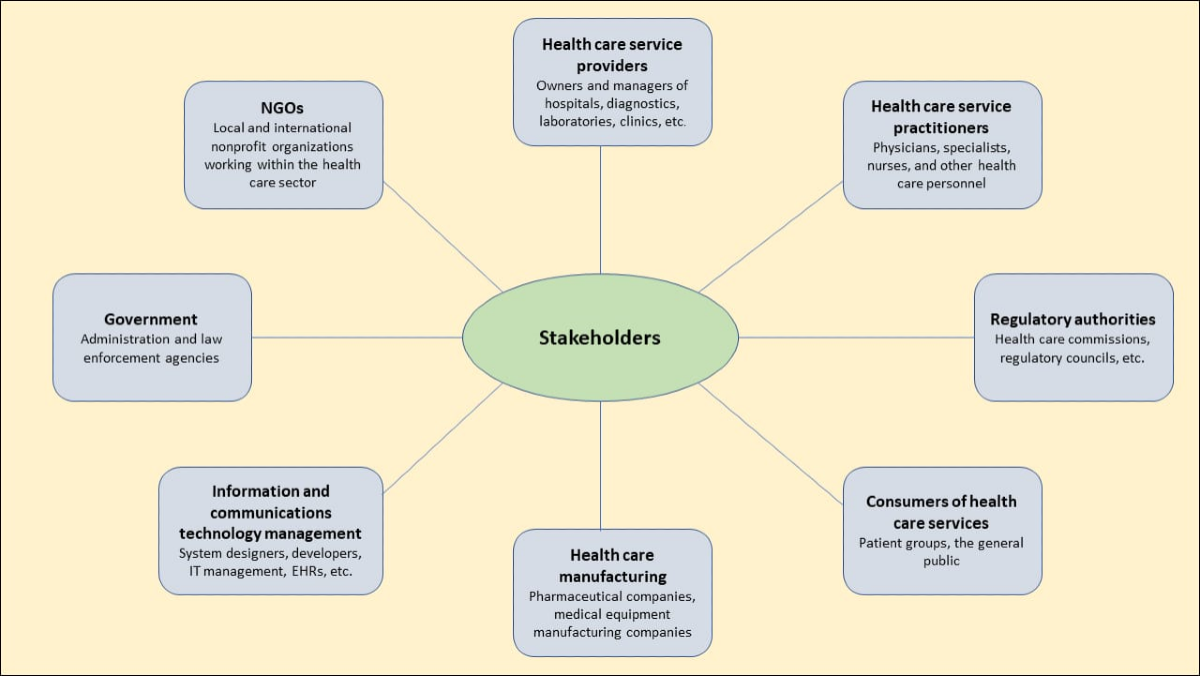
Stakeholders in health care management. EHR: electronic health record; IT, information technology; NGO: nongovernmental organization.

Stakeholders are considered to be among the important components of pandemic management processes. Usually, a pandemic requires greater participation of various stakeholders in health care management, as shown in Figure 1; this is applicable not only to health care practitioners but also to society as a whole [16]. Therefore, the stakeholders in pandemic times include society, where every individual shares the responsibility for containing the spread of infectious diseases. However, there is a need to define the roles and responsibilities of all stakeholders, including health care workers, research organizations, health care equipment manufacturers, and pharmaceutical companies, to prepare for pandemics [17]. Health care service providers and practitioners have the responsibilities of planning and delivering health care services, managing health care resources, etc. Regulatory authorities may need to monitor the health care service operations across various hospitals and formulate standards and regulations to be adopted by health care service providers and practitioners. Accordingly, manufacturing organizations should focus on meeting the growing needs for medicines, equipment, and other resources, while the information and communications management unit should focus on managing web-based health care operations and creating public awareness. Governments should monitor all health care operations and administer these at different levels, including cities, towns, and villages, in both public and private hospitals to prevent contamination and risk of infection. Pandemics may have a serious impact on people’s mental health. Anxiety, depression, and stress are illnesses that can result from fear about pandemics or under preventive measures such as isolation and social distancing [18,19]. Therefore, it is essential to provide support and awareness to the people. eHealth can be effective in this context, as support, services, and information can be remotely shared among people using various applications. Stakeholders usually rely on less timely and traditional sources of disease surveillance; therefore, there is a need for timely and reliable pandemic intelligence using effective communication technologies [20].

#### Operations Management

Operations management is another important area to be considered during pandemics. Available resources such as finance, medical supplies, equipment, devices, nurses, and practitioners need to be effectively managed, resulting in maximum efficiency of health care operations [21]. Large upfront investments in diagnosis, tests, and treatment were identified to result in the most efficient use of resources during pandemics [22]. In addition, pharmaceutical company operations related to research and development must be managed for maximum output. eHealth can play an important role in all these health-related operational activities by effectively disseminating quality information across the supply chain. In addition, operations such as diagnosis, disease surveillance, monitoring, tracing, and tracking can be effectively managed through mobile apps, which can limit the number of patients visiting hospitals and prevent the spread of infection.

#### Information Management

Information management is one of the most important aspects to be considered during pandemics. The successful management of a pandemic mostly relies on how information is managed and shared among stakeholders, which can help them take timely actions. The increasing number of myths related to COVID-19 is an example of poor information management, and rapid spread of incorrect information through social media platforms can result in serious damage, such as destruction of 5G towers in the United Kingdom [15] and drinking raw alcohol to prevent transmission of SARS-CoV-2 in Iran [23]. Effective monitoring and tracking and, most importantly, creating awareness about novel diseases and the precautions to be taken by the responsible authorities are key to the operational management and containment of infectious disease.

#### Strategy Development

The next set of factors focuses on the approach for developing strategies. These factors include strategic context; experiences from previous events and research; formulating a mission and objectives; identifying the target components; analyzing opportunities and gaps; developing strategies; implementation; monitoring and reviewing the approaches; and making necessary changes to approaches after deployment. Strategic context is related to the main area the approach is targeting. It may be related to containing infectious disease, reducing the spread of misinformation, or any other specific context related to pandemics [24]. Experiences from the past, such as approaches adopted by different governments to control pandemics such as SARS, MERS, Zika virus, and Ebola virus, can be used to identify the context. The mission statement is the foundation for developing the approach to managing pandemics. It must clearly define the purpose of the approach and instill a sense of motivation and hope among all the stakeholders [25] involved in containing pandemics. It should inspire and present a message of contributing to the delivery of the best health care services to people who are affected by pandemics through integrated clinical practice, education, research, and effective resource management to contain the pandemic.

#### Formulation of Objectives

The next step focuses on formulating the objectives. The objectives define the set of goals or activities that need to be achieved. These can include statements related to developing the workforce, developing research activities, improving health care services and delivery, etc. [25]. Next, the components that need to be targeted are identified; these can include different areas and activities related to health care management [24]. Based on the objectives, opportunities and gaps for achieving the specified objectives must be identified. Based on the identified components, objectives, and available resources, the necessary strategies for managing pandemics must be developed and implemented. The overall approach being adopted for managing pandemics must be monitored and evaluated. Based on the evaluation, the approach can be updated by making necessary changes in areas that are not effective. These guidelines can be used by different countries to develop eHealth strategies, as the basic components that must be considered are outlined. However, the success of the framework depends on how the strategies are formulated and implemented, which mainly involves the application of ICT to the health care framework.

**Figure 2:**
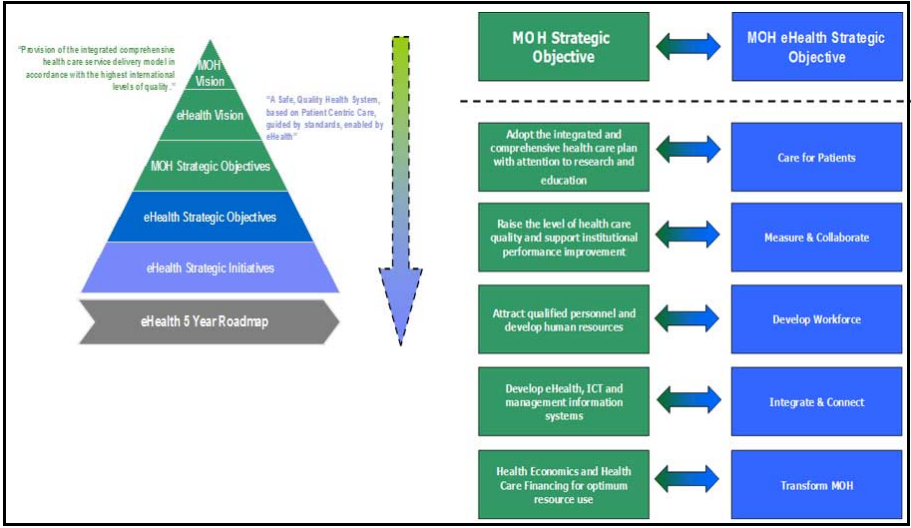
The National eHealth Strategy of Saudi Arabia. ICT: information and communications technology; MOH: Ministry of Health.

As part of this study, the National eHealth Strategy of Saudi Arabia (shown in Figure 2) is reviewed in the context of its applicability to managing pandemics. The framework provides the details and objectives of the approach, which are usually updated every five years. However, the current strategy is unclear and reflective in relation to various components, and it lacks a clear approach and process, supporting the findings of [1]. Necessary components such as information management, operations management, and stakeholders’ participation are not included in the framework. In addition, two important aspects, namely dissemination of legitimate information among all stakeholders (information accessibility) and preventing the circulation of wrongful information (information misuse), are not considered in the strategy. In this study, the strategy was further reviewed by health care experts in the context of pandemic management and the need for integrating additional components was assessed using a questionnaire-based survey, which is discussed in the next section.

## Methods

### Survey

The purposes of this study were to review the current National eHealth Strategy of Saudi Arabia in the context of managing pandemics and to propose an integrated eHealth framework that can be used in the general health care context as well as for managing pandemics. The questionnaire was divided into four components. The first component focused on questions related to the current National eHealth Strategy (four items); the second component focused on the key considerations in an eHealth framework (nine items); the third component focused on key factors in the eHealth framework (one item); and the fourth component focused on assessing the need for an integrated eHealth framework for managing pandemics such as COVID-19 in Saudi Arabia (two items). The items in the questionnaire were developed based on the components reviewed in the background study and the National eHealth Strategy toolkit provided by the WHO [24]. A 5-point Likert scale [26] was used to collect the responses to the questions. The participants could present their opinions on five scales (1, strongly agree; 2, agree; 3, neutral; 4, disagree; 5, strongly disagree) relating to each item in the questionnaire. The questionnaire was translated into Arabic by a professional translator. However, both English and Arabic versions were used in the process of data collection. The survey was designed using the Google Surveys platform, and survey links to the English and Arabic versions were created to invite the participants. A pilot study was conducted with six health care professionals (three practitioners, two nurses, and one manager). Based on the feedback from the participants, a few changes in the questionnaire formulation and multiple-choice options were made to address grammatical errors. In addition, the Cronbach alpha for all the items in the four components was identified to be >0.85, revealing good consistency. The English version of the survey is provided in Multimedia Appendix 1.

### Recruitment

Health care professionals were recruited using emails that included information about the purpose of the survey and the survey link. An additional note asking the participants to forward the email to their colleagues was included to increase the number of participants. Initially, using the portals at five hospitals (King Fahad Hospital in Jeddah, General Hospital in Medina, King Abdulaziz Medical City in Riyadh, King Khalid Ibn Abdul Aziz in HafarAlBatin, and King Khaled Hospital in Majma'ah), the email requesting participation in the survey was forwarded to all health care professionals working in these hospitals.

### Sampling

This study used purposive sampling, whereby a nonprobability sample was obtained based on the purpose and the objective of the study, which mainly focused on reviewing the current National eHealth Strategy and proposing a new integrated eHealth framework for managing pandemics. Accordingly, the survey link was initially forwarded to 257 health care professionals working at five hospitals in Saudi Arabia. Snowball sampling is an effective technique to reach a larger sample population in a short time. In this technique, existing study subjects are requested to recruit future subjects from among their acquaintances [27]. As a result of using snowball sampling (requesting participants to forward emails to their colleagues in other hospitals), a final sample of 350 participants was obtained. Of these participants, 23 were removed due to low-quality responses and incomplete data, and 11 more were removed because they did not work for health care–related organizations. As a result, 316 eligible participants completed the survey.

### Analytical Process

The survey was developed using the Google Surveys platform and was conducted from March 9 to April 6, 2020. Frequencies were calculated to analyze the collected data. The data were analyzed using four themes relevant to the study: review of the current National eHealth Strategy; objectives of the eHealth framework; importance of ICT; and need for an integrated eHealth framework in the context of managing pandemics in Saudi Arabia. The results are discussed in the following section.

## Results

The final sample size recruited for this study was 316 respondents. Among the sample of respondents, 221/316 of the participants were male (69.9%), and 95/316 (30.1%) were female. In terms of age, the majority of the participants were aged 35-44 years (147/316, 46.5%), followed by 25-34 years (110/316, 34.8%), 45-54 years (30/316, 9.5%), and 18-24 years (15/316, 4.8); only 4.4% (14/316) of the participants were older than 54 years. In terms of education, the majority of the participants had the qualification of a bachelor's degree (161/316, 50.9%). Numerous participants held a master’s degree (74/316, 23.4%) or doctorate (24/316, 7.6%), reflecting the level of expertise of the participants. In addition, 18% (57/316) of the participants had a diploma or high school qualification. In terms of profession, the sample was evenly distributed across the relevant experts. Of the participants, 26.3% (83/316) worked in administrative departments, while 23.4% (74/316) were nurses, 20.6% (65/316) were physicians, and 19.9% (63/316) were technical experts. In addition, 9.8% (31/316) of the participants were involved in other health care activities, such as research and development, pharmaceutical operations, and academics. In terms of work experience, 54.8% (173/316) of the participants had more than 10 years of experience, while 27.5% (87/316) had 5-10 years of experience. This indicates that 82.2% (260/316) of the participants had more than five years of experience, reflecting that the majority of the participants were reliable. In addition, 12.3% (39/316) of the participants had less than two years of experience, and 5.4% (17/316) had two to five years of experience. Table 1 shows the frequency distributions of these variables.

**Table 1:** Frequency distributions of key variables (N=316), n (%).

Variable		Value
**Gender**		
	Male	221 (69.9)
	Female	95 (30.1)
**Age (years)**		
	18-24	15 (4.8)
	25-34	110 (34.8)
	35-44	147 (46.5)
	45-54	30 (9.5)
	>54	14 (4.4)
**Education**		
	High school graduate or diploma	57 (18)
	Bachelor’s degree	161 (50.9)
	Master’s degree	74 (23.4)
	Doctorate	24 (7.6)
**Profession**		
	Physician	65 (20.6)
	Nurse	74 (23.4)
	Technical expert	63 (19.9)
	Administrator	83 (26.3)
	Other health care operation	31 (9.8)
**Work experience (years)**		
	<2	39 (12.3)
	2-5	17 (5.4)
	5-10	87 (27.5)
	>10	173 (54.8)

The next set of results focuses on reviewing the current national eHealth framework in Saudi Arabia. The effectiveness of the current framework in managing pandemics and infectious diseases was identified to be ineffective by the majority of the participants; 50.3% (159/316) of the participants disagreed and 8.9% (28/316) of the participants strongly disagreed with the statement that the current eHealth framework is effective, comprising nearly 60% of the total participants. Similarly, the statement that the current eHealth framework covers all major objectives of health care management received mixed responses. Although 40.2% (127/316) of the total participants disagreed and 7% (22/316) of the total participants strongly disagreed, a considerable number of participants (32%, 101/316) were neutral. However, the findings suggested that the current eHealth framework lacks a few objectives related to health care management. Similarly, considering the statement on inclusion of essential objectives for managing pandemics, 50% (158/316) of the participants disagreed, and 10.1% (22/316) of the participants strongly disagreed. Focusing on the need for revising the current framework, 53.8% (170/316) of the total participants strongly agreed and 16.5% (52/316) of the total participants agreed that the current framework should be revised, accounting for almost 70% of the total participants.

**Figure 3:**
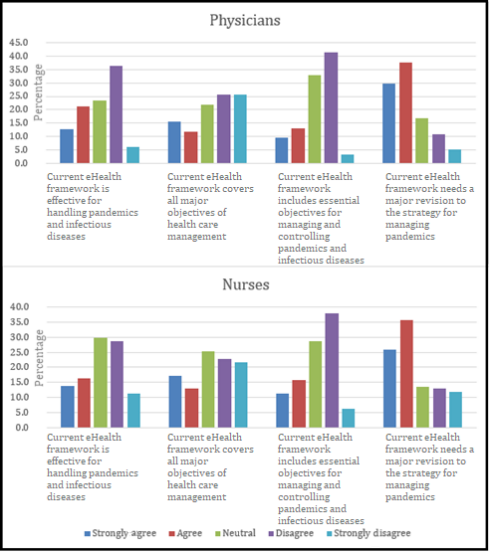
Comparison of the opinions of physicians and nurses regarding the current eHealth framework in Saudi Arabia.

In addition, 91.4% (289/316) of the total participants agreed that there is need for a new effective and efficient eHealth framework for Saudi Arabia. It can be observed from Figure 3 that the opinions about the current eHealth framework slightly differed between physicians and nurses. A slightly greater number of physicians than nurses stated the opinion that the current eHealth framework is ineffective for managing pandemics. However, the majority of both physicians and nurses stated the opinion that the current eHealth framework needs major revisions.

The next set of results (as shown in Table 2) focuses on the key considerations for developing a new integrated eHealth framework for managing pandemics in Saudi Arabia. In response to the statement that the new framework, in contrast to the existing framework, should focus on long-term objectives, 32.3% (101/316) of the participants strongly agreed with the statement, and 57.6% (182/316) of the participants agreed with it. Focusing on the distinction of services during normal and pandemic conditions may be useful to clearly outline the service delivery guidelines in these two conditions, which may clear any ambiguity regarding the operations and services. Accordingly, the idea of having different sets of objectives for the two conditions was supported by 94.6% (299/316) of the participants, among which 30.7% (97/316) strongly agreed with this statement and 63.9% (202/316) agreed with it. It is interesting to observe that 94% (297/316) of the total participants agreed that focusing on compatibility and integration is important. Accordingly, the need for inclusion of all stakeholders, including government, health care practitioners, businesses, and governments, and the need for clear roles and responsibilities of all stakeholders were supported by more than 85% of the total participants.

**Table 2:** Frequency distribution of key considerations related to the national eHealth framework of Saudi Arabia for managing pandemics (N=316), n (%).

Item	Strongly agree	Agree	Neutral	Disagree	Strongly disagree
The new framework should focus on long-term objectives	102 (32.3)	182 (57.6)	24 (7.6)	4 (1.3)	4 (1.3)
It should have different objectives relevant to managing health care services during pandemics	100 (31.7)	184 (58.2)	27 (8.5)	4 (1.3)	1 (0.3)
It should include regular health care objectives along with the objectives for controlling and managing pandemics	97 (30.7)	202 (63.9)	13 (4.1)	4 (1.3)	0 (0)
It should include all stakeholders: the public, health care practitioners, businesses, and government	109 (34.5)	166 (52.5)	33 (10.4)	7 (2.2)	1 (0.3)
It should specify clear roles and responsibilities for all stakeholders	121 (38.3)	175 (55.4)	17 (5.4)	0 (0)	3 (1.0)

The next set of results (as shown in Table 3) focuses on identifying the key factors to be considered in the framework for managing pandemics. The results demonstrate that public awareness, promotion of precautionary methods, dissemination of genuine information, increased stakeholder participation, increased reachability and accessibility of electronic services, improved communication, promotion of self-management and self-control approaches, formulation of new guidelines, and regular monitoring and updating of processes and approaches were identified to be the key factors in managing pandemics by the majority (>80%) of the total participants.

**Table 3:** Frequency distribution of key objectives to be included in the new eHealth framework (N=316), n (%).

Item	Strongly agree	Agree	Neutral	Disagree	Strongly disagree
Develop awareness-raising programs	124 (39.2)	168 (53.2)	18 (5.7)	4 (1.3)	2 (0.6)
Promote precautionary methods during pandemics	136 (43.0)	168 (53.1)	8 (2.53)	2 (0.63)	2 (0.6)
Make accurate information accessible to the public	115 (36.4)	159 (50.3)	31 (9.8)	8 (2.5)	3 (1.0)
Increase participation of all stakeholders at individual, community, and national levels	115 (36.4)	173 (54.8)	19 (6.0)	4 (1.3)	5 (1.6)
Increase the richness and reachability of electronic services	127 (40.2)	166 (52.5)	14 (4.4)	6 (1.9)	3 (1.0)
Heighten consciousness and improve communication	140 (44.6)	176 (51.3)	11 (3.5)	0 (0)	2 (0.6)
Promote self-management and self-control approaches among individuals	98 (31.0)	176 (55.7)	31 (9.8)	7 (2.2)	4 (1.3)
Formulate new guidelines for managing health care services during pandemics	106 (33.5)	182 (57.6)	23 (7.3)	3 (1.0.)	2 (0.6)
Review the framework at regular intervals and update the objectives	106 (33.5)	182 (57.6)	24 (7.6)	1 (0.3)	3 (1.0)

Focusing on the use of ICT, it may be considered that ICT is effective in managing information flow and streamlining operations to increase efficiency. Accordingly, in relevance to the importance of ICT for tracking and monitoring the spread of infectious disease, the majority of the participants (74.1%, 234/316) considered it to be important. In view of the rising number of pandemics in the past few decades, the importance and immediate need for an eHealth framework for Saudi Arabia were assessed in the context of COVID-19. The findings revealed that more than three-fourths of the participants (78.8%, 249/316) agreed that developing an integrated framework is very important. The results of the survey reflect the need for an integrated eHealth framework and various components that need to be considered to manage pandemics. The findings are accordingly discussed in the next section.

## Discussion

### Principal Findings

The results of this study clearly indicate that the current national eHealth strategy is ineffective for handling pandemics, as it does not incorporate all the necessary components and objectives for managing pandemics using eHealth strategies. In addition, 89.9% (284/316) of the survey participants were in favor of developing an integrated eHealth framework for managing pandemics. Focusing on these gaps, the necessary components of the framework were identified based on the findings (Table 2, Table 3) related to the key considerations and key factors to be included in the framework for managing and controlling pandemics. Firstly, it is important to determine the strategic context. The strategic context describes the priorities and challenges that eHealth can address. This context can be developed by reviewing the health statistics of the population, current health strategy, priorities, and goals. As identified from the key considerations (Table 3), 88.9% (281/316) of the participants stated that long-term objectives should be considered. As pandemics can have long term impacts [28,29], the framework should focus on both short-term (as adopted in the current National eHealth Strategy) and long-term objectives. In addition, it is necessary to consider and review the current health care system. Accordingly, 93% (294/316) of the total participants stated that the objectives related to general health care and health care services during pandemics must be considered. In addition, the priorities and goals must be revised according to the situations that arise during pandemics. In addition, past trends and experiences in dealing with pandemics can be used as a valuable source of information for setting the context, priorities, and goals [29].

A vision statement presents a long-term objective, while a mission statement considers short-term objectives for achieving the vision. Managing and controlling epidemics requires short-term objectives that are effective in delivering health care services and preventing the spread of infectious disease using eHealth approaches. Based on the mission statement, the next step is to assess the components required to achieve the mission. These components can also be considered as building blocks for achieving the mission; they include leadership and governance, strategy and investment, services and applications, standards and interoperability, infrastructure, legislation, policy and compliance, and the workforce [24]. The leadership, standards and interoperability, investment, policy, and compliance components are ensured by governments and health care ministries when establishing the mission and overseeing all health care operations. Although infrastructure, services, and applications are related to the ICT environment, all other components can be considered as enablers in creating the environment for managing pandemics.

**Figure 4:**
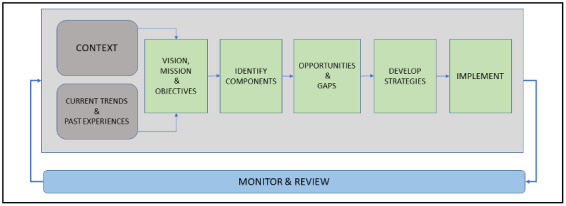
Approach for developing eHealth strategies during pandemics.

The next step is to analyze the opportunities, gaps, risks, and barriers for creating an eHealth environment for the components identified. While the opportunities suggest possible eHealth solutions and applications relevant to components, gaps can be identified in relation to services and infrastructure [29-32]. For instance, opportunities for collaboration between research and development and health care units such as hospitals, physicians, and pharmaceutical companies can be achieved by using a common electronic platform or portal for sharing information related to diseases, such as symptoms, diagnosis factors, effects of various medicines, and need for medicines and medical equipment. Lack of effective collaboration between these components can create risks and gaps related to the delivery of health care services; this can be assessed from the recent outbreak of COVID-19, where shortages of medical equipment and vaccines and a lack of information are clearly evident in various regions [32,33]. Based on the assessment of opportunities and gaps, strategies can be developed and implemented. As identified from the findings (Table 3), strategies such as promotion of precautionary methods, creating awareness, promotion of self-management and self-control practices, and increasing accessibility to genuine information were considered to be highly important during pandemics. One of the most important aspects in the process of developing strategies during pandemics is rapid monitoring and review of the overall development and implementation process. Accordingly, the whole approach is presented in Figure 4.

**Figure 5:**
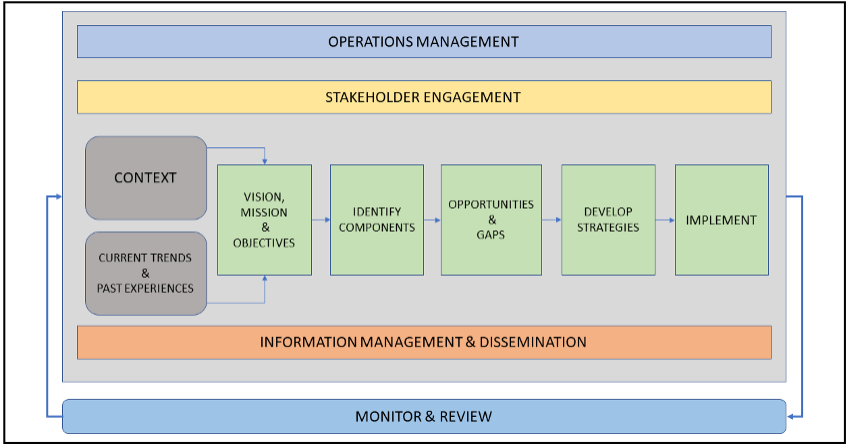
Proposed integrated eHealth framework for pandemic management.

A few components must be managed throughout the process, including operations management, stakeholders' engagement, and information management, as shown in Figure 5, representing an integrated eHealth framework for managing and controlling pandemics in Saudi Arabia. Health care operations during pandemics involve service delivery and administration of health care activities; moreover, the scope of operations is increased by engaging all the stakeholders and their respective activities. This process requires high-level leadership and support, an appropriate governance structure, a multidisciplinary team, and an agreed timeline and resources for completing the tasks with maximum efficiency in the minimum amount of time [24]. For instance, the manufacturing of masks, testing kits, and medicines such as hydroxychloroquine is being implemented on a “wartime” footing in different countries for managing and controlling COVID-19 [34,35], and lack of effective leadership and strategy may result in various risks and challenges for controlling pandemics [36,37]. This represents a collaborative effort under the leadership of governments with various stakeholders, including physicians, pharmaceutical companies, and manufacturers of testing kits, operating with maximum efficiency to address the needs of health care systems during a pandemic.

Stakeholder engagement is another important component that must be managed during pandemics. The engagement process should focus on government's leadership roles, such as overseeing the engagement of all stakeholders; identifying different stakeholder groups, such as health care professionals, the public, pharmaceutical companies, medical equipment manufacturers, administrators, managers, and all other personnel involved with and related to health care activities; developing an approach for managing these groups; and defining the points of consultation and dissemination of information across all stakeholder groups. Some major stakeholder groups, as analyzed in [24], include decision-makers, key influencers, engaged stakeholders, broader stakeholders, and the general public. Leaders should ensure supportive and constructive engagement of all stakeholders during a pandemic, involving everyone in the process of managing and controlling the pandemic. Great benefits of ICT in health care management can be realized during pandemics. ICT can help prevent the spread of misinformation and can create awareness about infectious diseases, promote precautionary methods, provide accessibility of health care services through enhanced web-based or mobile applications, disseminate information and services in remote areas, and promote self-management and self-control practices during pandemics. All these factors were rated to be highly important by the majority of the survey participants (Table 3).

Thus, the three additional components are integrated with the strategy development components to form an integrated eHealth framework for managing and controlling pandemics. The process of review and monitoring is accordingly applied to all the components in the integrated eHealth framework, as shown in Figure 5. Accordingly, all the essential components of an eHealth strategy for managing pandemics are identified and integrated.

### Limitations

In this study, various eHealth components required for managing and controlling pandemics were identified. However, functions within these components may differ from an application perspective in different regions. There is a need for clear definitions and explanations of these functions; these are not presented in this study, as it focused only on identifying the key components at the national level. In addition, the framework was developed specifically in a Saudi Arabian context based on a review of the current National eHealth Strategy; thus, it may not be applicable to other countries. However, it was ensured that the selection of components was generalized and focused on the necessity for health care management during pandemics.

### Implications

Both theoretical and practical implications can be derived from this study. The literature review and the proposed framework can be used by researchers as a concept for developing and evaluating various strategies during pandemics, such as the lockdown strategy during the COVID-19 pandemic. In addition, the proposed framework offers valuable information for academicians and researchers regarding eHealth components and approaches for developing and implementing eHealth strategies. Practical implications include the consideration of the proposed framework by the Saudi Arabian government and the Ministry of Health in developing and implementing effective eHealth strategies during pandemics by relating the eHealth strategies to the various components proposed in the framework.

### Future Research

The framework proposed in this paper is specific to Saudi Arabia. Future research will focus on evaluating the framework in the context of Saudi Arabia. Although the framework was designed in the context of Saudi Arabia, it may be applicable to other regions with similar operational health care infrastructures and strategies. Therefore, future research may focus on validating the framework in similar countries in the Middle East and in other developing countries. In addition, both qualitative and quantitative methods can be used in the evaluation of the framework, which may lead to the collection of various types of data from which various inferences can be made. The author also proposes to extend the framework to various health care operations and the involvement of stakeholders (roles and responsibilities) using the approach of collective intelligence, where active engagement of all global stakeholders is considered to overcome the challenges of pandemics.

### Conclusion

This study reviewed the current National eHealth strategy of Saudi Arabia; the review revealed various limitations and drawbacks in relation to health care management during epidemics. Accordingly, a survey instrument was administered to health care professionals to identify the necessary eHealth components and an approach was described for developing and implementing the strategies in relation to the identified components. The proposed framework is considered to be effective and efficient, as it is designed to be used for managing both general health care services and essential health care services during pandemics. This study proposes the main components of an eHealth framework for managing and controlling pandemics. Future work will focus on evaluating the identified components and identifying the necessary functions related to each component that are necessary during pandemics.

## Appendix

Multimedia Appendix 1 Survey questions.

## Abbreviations

ICT: information and communications technology
MERS: Middle East respiratory syndrome
SARS: severe acute respiratory syndrome
WHO: World Health Organization
