# Supplementary material for: Applying eHealth for Pandemic Management in Saudi Arabia in the Context of COVID-19: Survey Study and Framework Proposal
Source: JMIR Med Inform. 2020 Nov 26;8(11):e19524. doi: 10.2196/19524 (PMC7695544; doi:10.2196/19524)
Supplement: Multimedia Appendix 1 [file medinform_v8i11e19524_app1.docx]

Survey for reviewing E-Health Strategy in Saudi Arabia in relation to Epidemics

This survey is aimed at investigating the effectiveness and efficiency of National W-Health Strategy, and its effectiveness in dealing with epidemics such as COVID-19. Roadmap for E-Health strategy in Saudi Arabia is developed every five years (short-term), and it is aligned with strategic objectives. Please review the current E-health strategic framework and strategic objectives in the following figures, and kindly answer the relevant questions in the survey. Participants anonymity will be maintained and it is ensured that the data obtained from the survey will be used for academic purpose only.


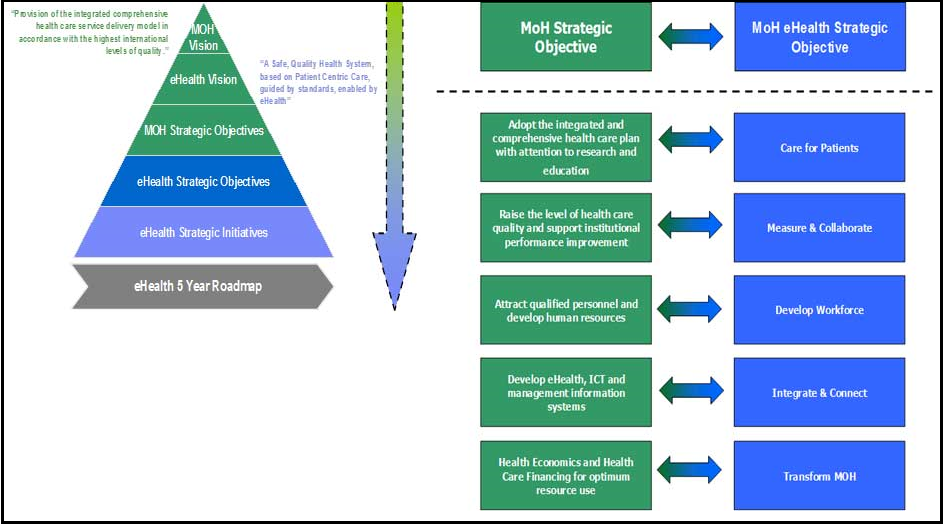


PART- A: PARTICIPANTS DETAILS

1. NAME

2. AGE: <15 years; 15-24 years; 25-34 years; 35-44 years; 45-54 years; >54 years

3. GENDER: M/F

4. EDUCATION: School/Bachelor Degree/Masters/Ph.D./Others

5. Do you work in healthcare related organization?

- Yes
- No

6. Profession:

- Physicians
- Nurse
- Technical Experts
- Administrative
- Others

7. Work Experience: <2 years; 2-5 years; 5-10 years; >10 years

PART B: Framework Review

8. Please rate the following on a scale of (1: Strongly Disagree; 2: Disagree; 3: Neutral; 4: Agree; 5: Strongly Agree)

- Current E-Health Framework is effective for handling epidemic/infectious diseases.
- Current E-Health Framework covers all major objectives of healthcare management
- Current E-Health Framework includes essential objectives for managing and controlling epidemic/infectious diseases
- Current E-Health Framework needs a major revision on the strategy for managing epidemics

9. Do you think there is need to develop a new E-health framework in specific for managing epidemics?

- Yes
- No

PART C: New Framework

10. Please rate the following statements related to new E-health framework on a scale of (1: Strongly Disagree; 2: Disagree; 3: Neutral; 4: Agree; 5: Strongly Agree)

- New Framework should focus on long-term objectives
- It should have different objectives relevant to managing healthcare services during epidemics
- It should include regular healthcare objectives along with the objectives for controlling and managing epidemics
- It should include all stakeholders: Public, Healthcare Practitioners, Businesses, and Government
- It should specify clear roles and responsibilities for all stakeholders

11. Please rate the following additional objectives for new E-health framework on a scale of (1: Strongly Disagree; 2: Disagree; 3: Neutral; 4: Agree; 5: Strongly Agree)

- Develop Awareness raising Programs
- Promote the precautionary methods during epidemic
- Make genuine information accessible to public (FAKE INFO & INF ACCESSIBILITY)
- Increased participation of all stakeholders at individual, community, and national levels in controlling and managing epidemic
- Increasing richness and reachability of E-Services
- Heightened consciousness and improved communication
- Promoting Self-management and self-control approaches among individuals
- Formulating new guidelines for managing healthcare services during epidemic [ (people, technological, informational, and financial) management, and operations management.]
- Review of the framework at regular intervals and updating the objectives

12. How do you rate the importance to deploying ICT services to identify and track the spread of infectious disease across the country?

- Highly important
- Important
- Neutral
- Not important
- Not necessary

13. How do you rate the importance and need for having an E-health framework for managing epidemics in light of recent COVID-19.

- Highly important
- Important
- Neutral
- Not important
- Not necessary
